# Quality of transurethral resection of bladder tumor procedure influenced a phase III trial comparing the effect of KLH and mitomycin C

**DOI:** 10.1186/s13063-017-1843-5

**Published:** 2017-03-14

**Authors:** Narasimha N. G. Prasad, Shammana N. Muddukrishna

**Affiliations:** 1 Department of Mathematical and Statistical Sciences, University of Alberta, T6G 2G1, Edmonton, AB Canada; 2 Biosyn Corporation, 5939 Darwin Court, Suite 114, Carlsbad, CA 92008 USA

## Abstract

**Background:**

Retrospective analysis of Center effect of the multi-center trial conducted to compare Immucothel (KLH Immunotherapy drug product) with Mytomycin-C (MM) concluded that efficacy evaluation of the drug product may be impacted by physician’s subjective performance of Transurethral resection of bladder tumor (TURBT).

**Methods:**

A randomized trial was performed in 18 hospitals (clinical centers) and a total of 553 recruited, 283 patients under KLH arm and 270 patients under MM. An initial statistical analysis of efficacy comparisons between KLH and MM based on log-rank test performed for each center (hospital) showed 6 hospitals out of 18 hospitals a *p*-value of <0.05 and remaining 12 hospitals showed a *p*-values of >0.05. No association was observed between number of patients analysed and the associated p-values across hospitals. Final statistical analyses were carried out under each drug product using Kaplan-Meier survival analysis along with log-rank test after combining all eligible patients data for 6 hospital group and 12 hospital group.

**Results:**

Median recurrence free survival (RFS) times (in weeks) showed statistical significance (*p*-value = 0.03) between two groups of hospitals under KLH arm, while similar median values showed no statistical significance (*p*-value = 0.05).

**Conclusion:**

Center effect with respect to median RFS values under KLH was more pronounced than under MM. Under the presence of such center effect, for reasons other than product related effects, concluding superiority of one drug product over another may create confounding bias conclusions in multi-center clinical trials. In the above cited clinical trial study, physician’s prior experience on TURBT might have contributed to center effect in examining efficacies of KLH and MM. Similar observation was also noted in the literature on studies dealing with TURBT and in other clinical studies.

**Trial registration:**

Data set used in this study is based on previously documented clinical trial in the literature: See (Lammers et al., J Clin Oncol 30:2273–9, 2012) and Acknowledgments.

**Electronic supplementary material:**

The online version of this article (doi:10.1186/s13063-017-1843-5) contains supplementary material, which is available to authorized users.

## Background

Intravesical bacillus Calmette-Guérin (BCG) instillation remains the standard treatment among intravesical anticancer therapies after transurethral resection (TURBT) for bladder cancer, to suppress disease recurrence and progression [1]. Since the toxicity of BCG is a limitation in some patients, other alternative intravesical chemotherapeutic agents have been developed. One such cytostatic agent is mitomycin C (MM). IMMUCOTHEL® (Keyhole Limpet Hemocyanin (KLH) Immunotherapy drug product, biosyn, Fellbach, Germany) is an approved drug product for the prevention of bladder carcinoma recurrence after TURBT and after failure of other established therapies, like BCG and MM.

A randomized phase III trial based on 553 patients (283 patients under KLH and 270 patients under MM), with intermediate- and high-risk non-muscle-invasive bladder cancer (NMIBC) without carcinoma in situ has been performed and the study details are published [2]. The multicenter trial spanned over 18 clinical centers (hospitals). In the phase III trial study patients were randomized into the KLH arm or the MM arm after TURBT. In the KLH arm, patients received 1 mg KLH preimmunization followed by instillations with 20 mg KLH once per week for 6 weeks and once per month for 10 months for a total targeted 16 instillations as part of the KLH treatment. In the MM arm, patients received 40 mg MM once per week for 4 weeks and followed by five monthly instillations, and then at 9 and 12 months for a total targeted 11 instillations. The efficacy comparison between KLH and MM was based on median recurrence-free survival (RFS) and recurrence rate [2].

The present work focus is on exploratory evaluation of a treatment-by-centre interaction effect through statistical significance tests for treatment effects within each centre based on ICH Guidance [3].

## Material and Methods

Patient demographic and clinical characteristics of the multicenter phase III clinical trial have already been published by Lammers et al. (Table 1) [2]. Initial analysis of the 18 individual hospitals’ data was performed using the by Kaplan-Meier method and hazard ratio (HR) using the Cox PH model. The statistical analysis is summarized in Table 1.

**Table 1:**
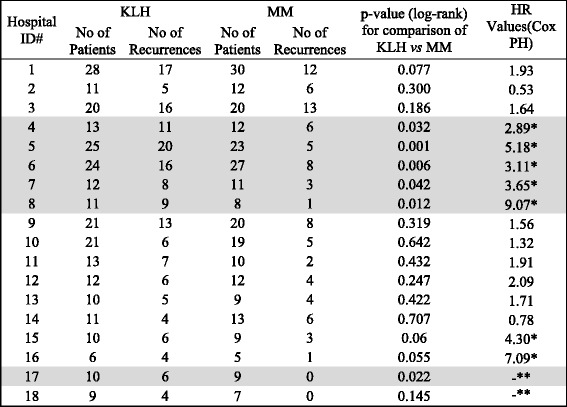
Summary of recurrences, *p* value (log-rank) and hazard ratio (HR) values (Cox PH) for 18 trial centers

Hospitals with *p* < 0.05 highlighted in gray

*HR value larger than 2.26 (over all HR value based on all 18 hospitals)

**HR value not available since no recurrences in the mitomycin C group

*KLH* Keyhole Limpet Hemocyanin, *MM* mitomycin C, *TURBT* transurethral resection of bladder tumor

The overall HR (18 centers) is 2.26 as reported by Lammers et al. [2] and confirmed in our analysis. The hospital grouping based on HR and *p* values shows a fairly good overall correlation. All the hospitals (5 out of 5, 100%) with *p* < 0.05 have a HR value greater than 2.26 and majority of hospital (9 out of 11, 81%) with *p* > 0.05 have a HR value less than 2.26. The HR values for two hospitals, 17 and 18, are not reported as there was no recurrence in MM treatment arm (Table 1). In view of these observations subsequent analysis was performed using hospital grouping based on *p* values.

In Table 1, the *p* value for six hospitals (shaded in gray) was statistically significant; *p* value < 0.05. Those six centers were designated as group 1. The remaining 12 hospitals had a *p* value > 0.05 and were pooled in group 2. The patient demographic and clinical characteristics of all patients for group 1 and group 2 are summarized in Table 2.

**Table 2:** Patient demographic and clinical characteristics of all patients within the two hospital groups

Characteristic	KLH				MM				Total	
	Group 2		Group 1		Group 2		Group 1		KLH	MM
	*N*	%	*N*	%	*N*	%	*N*	%		
Number of patients	172	64.4	95	35.6	166	64.8	90	35.2	267	256
Sex										
Male	141	82	77	81.1	133	80.1	77	85.6	218	210
Female	31	18	18	18.9	33	19.9	13	14.4	49	46
Mean age in years	37.6		36.8		35.6		47.9			
Tumor status										
Primary	100	58.1	44	46.3	102	61.4	44	48.9	144	146
Recurrent	72	41.9	51	53.7	64	38.6	46	51.1	123	110
Previous treatment in patients with recurrent tumors at baseline										
Current single post-TURBT intravesical chemotherapy	2		3		1		1		5	2
Previous single post-TURBT intravesical chemotherapy	8		2		2		1		10	3
Local adjuvant intravesical therapy	20		11		12		15		31	27
Other therapy	3								3	3
No therapy other than TURBT	39		35		49		29		74	78
Tumor stage										
pTa	132	76.7	84	88.4	113	68.1	71	78.9	216	184
pT1	40	23.3	11	11.6	53	31.9	19	21.1	51	72
Tumor grade										
1	54	31.4	34	35.8	47	28.3	32	35.6	88	79
2	95	55.2	53	55.8	87	52.4	47	52.2	148	134
3	23	13.4	8	8.4	32	19.3	11	12.2	31	43
Number of tumors										
Single	60	34.9	29	34.9	57	34.3	26	28.9	89	83
Multiple	112	65.1	66	69.5	108	65.1	64	71.1	178	172
Unknown					1					1

*KLH* Keyhole Limpet Hemocyanin, *MM* mitomycin C, *TURBT* transurethral resection of bladder tumor

Group 1 represents six hospitals, which show a statistically significant difference (*p* value < 0.05) between the two treatment groups. The data in group 1 consists of 95 patients in the KLH treatment arm and 90 patients for MM. Group 2 represents the remaining 12 hospitals, which displayed no statistically significant difference (*p* value > 0.05). Group 2 includes 172 patients for KLH and 166 for MM.

The median RFS and recurrence rates for KLH and MM were derived by Kaplan-Meier survival analysis. Comparison of median RFS using the log-rank test was performed for the same drug product (KLH or MM) between group 1 and group 2.

## Results and Discussion

### Significant differences between trial centers

The analysis of the 18 individual trial centers showed significant differences for six hospitals (*p* < 0.05) for both treatment arms. Therefore, those six centers were designated as group 1 and the remaining 12 hospitals were pooled in group 2 (Table 1). In total, 95 patients (35.6%) received KLH and 90 (35.2%) patients were treated with MM in group 1. In group 2, 172 patients (64.4%) were treated with KLH and 166 (64.8%) received MM (Table 3). Patient demographic and clinical characteristics are shown in Table 2. The data suggests that the distribution of patients for KLH and MM arms is similar between group 1 and group 2.

**Table 3:** Frequency of subjects and number of recurrences in the two hospital groups

Treatment received	Hospital group^a^	Total *N*	*N* of events (# of recurrences)	Recurrences in [%]
KLH	Group 1	95	70	74
	Group 2	172	93	54
	Overall	267	163	61
Mitomycin C	Group 1	90	23	26
	Group 2	166	64	39
	Overall	256	87	34

^a^Group 1 = 6 hospitals, Group 2 = 12 hospitals

*KLH* Keyhole Limpet Hemocyanin

### Recurrence rate and median RFS were significantly different within treatment arms

The recurrence rate and median RFS for KLH and MM in group 1 and group 2 are summarized in Table 4. For the KLH treatment arm, 74% (70 out of 95 patients) developed a recurrence in group 1 as compared to 54% (93 out of 172 patients) in group 2 (*p* value = 0.03, chi-square test). For the MM treatment arm, 26% (23 out of 90 patients) developed a recurrence in group 1 as compared to 39% (64 out of 166 patients) in group 2 (*p* value = 0.05, chi-squared test).

**Table 4:** Recurrence rate and median recurrence-free survival (RFS) for Keyhole Limpet Hemocyanin (KLH) and mitomycin C (MM) for group 1 and group 2

Treatment received	Hospital group^a^	Recurrence rate (%)	Median (weeks)	95% confidence interval	
				Lower bound	Upper bound
KLH	Group 1	74	84	64	103
	Group 2	54	139	100	178
Mitomycin C	Group 1	26	N/A^b^	N/A^b^	N/A^b^
	Group 2	39	297	152	442

^a^Group 1 = 6 hospitals, Group 2 = 12 hospitals

^b^Not attainable

The median RFS for KLH in group 1 is 84 weeks compared to 139 weeks for median RFS for KLH in group 2. The median RFS for group 1 is significantly lower than group 2 (*p* = 0.004, log-rank test). Similarly, the median RFS for group 1 is not attained for MM compared to a median RFS value of 297 for group 2 (approaching significance *p* = 0.05, log-rank test).

Similar results for recurrence rate and medium RFS were obtained when the hospital grouping was performed based on HR values (low <2.26 and high >2.26) (details not reported; se﻿e Additional files 1 and 2).

These results imply that the same drug product has better efficacy in one group compared to the other. Logically, this would be an unlikely scenario and suggests that other center-related effects may be involved in this study.

### Significant influence of prior TURBT in multi-center trial

The study design called for TURBT prior to randomizing the patients into each treatment arm. Existing medical literature data suggests that outcome of TURBT with regard to recurrence is significantly influenced by the operative experience of the surgeon [4–10]. The results of this analysis provide additional evidence to the referenced existing medical literature data. The observed differences between group 1 and group 2 hospitals were possibly due to differences in the operative experience of the surgeons.

What are the possible approaches that can be taken in the design of clinical trials to overcome this TURBT-related variability in evaluating new drug product efficacies? One approach could be recruiting patients who have undergone at least one TURBT procedure. In this regard, we have performed a subgroup analysis of patients receiving 16 KLH instillations or 11 MM instillations and who had prior recurrent tumors (*n* = 156; KLH = 67 and MM = 89). The results showed that the percentage of recurrences for KLH-treated patients was 54% while the percentage of recurrence for MM treated patients was 38%. Statistical evaluation of product efficacy (chi-squared test) indicated that the two groups are not significantly different (*p* = 0.107).

Further supporting data has been reported by Lammers et al. [2]. Subgroup analysis performed for patients with recurrent disease and a history of intravesical treatments, the number of patients with recurrences and time to recurrence was not statistically different for the KLH and MM treatment groups.

### Different efficacy profile for KLH and mitomycin C exacerbate influence of TURBT on therapy outcome

KLH is a nonspecific immunotherapeutic agent and unlike chemotherapeutics, such as mitomycin C, the immunotherapeutic effect to take hold requires some lead time to produce a positive outcome in patients and, as such, the treatment course includes 16 instillations over a 12-month time period. KLH (IMMUCOTHEL®) is approved as a second-line treatment. Therefore, subgroup analysis was performed for patients with recurrent disease and a complete treatment course who had received an additional treatment after TURBT for prior tumor. The number of patients with recurrences (45% versus 52%) and time to recurrence (235 versus 98 weeks) was not statistically different for the KLH and MM treatment groups (*p* ~ 1.0, respectively, *p* = 0.549) (Table 5).

**Table 5:** Recurrence rate and median recurrence-free survival (RFS) for Keyhole Limpet Hemocyanin (KLH) and mitomycin C (MM) in patients with recurrence and complete treatment course who received additional treatment after transurethral resection of bladder tumor (TURBT) for prior tumor (KLH second-line treatment)

	Recurrence rate (%)	Median (weeks)	95% confidence interval	
			Lower bound	Upper bound
KLH	48	235	35	435
Mitomycin C	52	98	75	121
Overall	50	210	69	351

In general, it appears that the recurrence rate between institutions or multiple clinical studies for bladder cancer patients treated with any of the currently used drug products (BCG and MM included) has a large variability [2, 4, 9].

## Conclusion

The results of the prospective, randomized, multicenter, phase III clinical trial, “Intracutaneous and Intravesical Immunotherapy with Keyhole Limpet Hemocyanin Compared with Intravesical Mitomycin in Patients with Non-Muscle-Invasive Bladder Cancer,” have been published [2]. The authors concluded that KLH has a different safety profile and is inferior to MM in preventing non-muscle-invasive bladder cancer.

This retrospective analysis of the same clinical data focused on evaluating center effects on product efficacy. From the statistical analysis of the individual centers, we identified two sets of hospital groups based on the *p* values (*p* < 0.05 and *p* > 0.05). The results indicate that the majority of the hospitals showed no statistical difference between KLH and MM with respect to median RFS. This suggests the presence of center effects in this clinical trial as evidenced by statistical comparison of the same drug product (KLH or MM) between the two hospital groups. The conclusion of this data would be that the same drug product is superior to itself between the two centers. As this is an illogical conclusion; among other possible determining factors, the TURBT procedure is suspected to be the main contributing factor. The patient data summary in Tables 1 and 2 suggests that there is no selection bias arising due to clinical and demographic patient characteristics. Such selection bias would normally be a prime determining factor but is not present in this well-designed clinical trial. Similar suspicions have been raised by other reports as it relates to variable observed recurrence rates between institutions. This report specifically expands on that concept to show that it has an effect on the efficacy determination of drug products.

This suggests that when performing new drug efficacy evaluation studies involving surgery as a prerequisite step, as is the case with bladder cancer, the operative experience of the surgeon [10, 11] is an additional important factor for consideration in designing future clinical studies.

In view of such differences in the efficacy of the same product used on patients in two groups of hospitals, proper care and procedures need to be taken in generalizing product efficacies. As suggested by Richsterstetter et al. [8] and Brausi et al. [9], suitable statistical procedures need to be utilized to account for interaction effect between product efficacy and multicenter effects before generalizing results of the clinical study. This additional confounding effect has to be taken into consideration when designing clinical trials with new drug products involving surgery as a prerequisite treatment step.

Once a product is approved, surgeons optimize surgical technique for a given drug product to provide improved clinical outcomes for their patients. Such modifications may be done unintentionally. As a result, when surgeons perform clinical studies using new product candidates, there may be an inherent bias in their surgical techniques toward their preferred choice of drug product.

## Additional files

Additional file 1: Table S1. Frequency of subjects and number of recurrences in the two hospital groups using hazard ratio (HR) values reported in Table 1. **Table S2.** Recurrence rate and median recurrence-free survival) (RFS) for Keyhole Limpet Hemocyanin (KLH) and mitomycin C (MM) for group S1 and group S2. **Table S3.** Hazard ratio for the same drug product KLH/MM for group S1 relative to group S2. (PDF 148 kb)

Additional file 2: Supplementary material. (PDF 165 kb)

## Abbreviations

BCG: Bacillus Calmette-Guérin
KLH: Keyhole Limpet Hemocyanin
MM: Mytomycin C
RFS: Recurrence-free survival
TURBT: Transurethral resection of bladder tumor
