# Supplementary material for: Quality of transurethral resection of bladder tumor procedure influenced a phase III trial comparing the effect of KLH and mitomycin C
Source: Trials. 2017 Mar 14;18:123. doi: 10.1186/s13063-017-1843-5 (PMC5351261; doi:10.1186/s13063-017-1843-5)
Supplement: Supplementary file 1 — Frequency of subjects and number of recurrences in the two hospital groups using hazard ratio (HR) values reported in Table 1. Table S2. Recurrence rate and median recurrence-free survival) (RFS) for Keyhole Limpet Hemocyanin (KLH) and mitomycin C (MM) for group S1 and group S2. Table S3. Hazard ratio for the same drug product KLH/MM for group S1 relative to group S2. (PDF 148 kb) [file 13063_2017_1843_MOESM1_ESM.pdf]

Additional file 1

Table S1: Frequency of subjects and number of recurrences in the two hospital groups using HR values reported in Table 1.				
Treatment actual received	Hospital group[1]	Total N	N of events (# of recurrences)	Recurrences in [%]
KLH	Group S1	101	74	73
	Group S2	147	79	54
	Overall	248	153	62
Mitomycin C	Group S1	95	27	28
	Group S2	145	60	41
	Overall	240	87	36

[1]Group S1 (High HR values)= 7 Hospitals, Group S2 (Low HR values) =9 Hospitals.
Hospital group 17 & 18 not included (HR not available)

Table S2: Recurrence Rate and Median RFS for KLH and MM for Group S1 and Group S2					
Treatment actually received	Hospital group[1]	Recurrence Rate (%)	Median (weeks)	95% Confidence Interval	
				Lower Bound	Upper Bound
KLH	Group S1	73	86	64	99
	Group S2	54	135	104	210
Mitomycin C	Group S1	28	N/A[2]	N/A[2]	N/A[2]
	Group S2	41	297	123	N/A[2]

[1]Group S1= 7 Hospitals, Group S2=9 Hospitals
[2]Not Attainable

Table S3: Hazard Ratio for the same drug product KLH/MM for Group S1 relative to Group S2				
Treatment	HR	p-value (z test) for testing HR=1	95% Confidence Interval	
			Lower Bound	Upper Bound
KLH	1.55	0.005	1.14	2.11
Mitomycin C	0.6	0.026	0.38	0.94
