# Supplementary material for: Quality of transurethral resection of bladder tumor procedure influenced a phase III trial comparing the effect of KLH and mitomycin C
Source: Trials. 2017 Mar 14;18:123. doi: 10.1186/s13063-017-1843-5 (PMC5351261; doi:10.1186/s13063-017-1843-5)
Supplement: Supplementary file 2 — Supplementary material. (PDF 165 kb) [file 13063_2017_1843_MOESM2_ESM.pdf]

Additional file 2 Supplementary Material.

Hospital grouping and analysis based on hazard ratio (HR)

The hazard ratio (HR) for each hospital using Cox PH model, are computed with RFS as time variable and treatment arm (KLH and MM) as covariate. Results are summarized in Table 1. For hospital number 17 and 18, the HR values are not available since no recurrences were observed within study time in one of the treatment arm (MM). The overall HR value for the 18 hospitals is 2.26. The hospital grouping was performed based on low HR (<2.26) and high HR (>2.26) for 16 hospitals, hospitals 17 and 18 are excluded in this analysis. High HR group is designated as Group S1 and low HR group is designated as Group S2.

Results and Discussion:

Table S1 summarizes frequency of subjects and number of recurrences in Group S1 and Group S2 . The data in Group S1 consists of 101 patients in the KLH treatment arm and 95 patients for MM. Group S2 includes 147 patients for KLH and 145 patients for MM. In KLH arm 73% of patients in all hospitals in Group S1 had recurrences during the study period as compared to 54% in Group S2. In MM arm the rate was 28% for Group S1 compared to 41% for Group S2. These recurrence rates for the two Group S1 and S2 are similar to Group 1 and Group 2 (based on log rank p-value grouping, Table 3).

Table S2 summarizes the results of recurrence rate and median RFS. The median RFS estimates and confidence interval are based on Kaplan-Meier method. For KLH the median RFS estimate for Group S1 had a smaller value (86 weeks) than the corresponding value for Group S2 (135 weeks). For MM arm median RFS estimate for Group S2 is 297 weeks where as corresponding value for Group S1 is not available. These values for the Group S1 and S2 are similar to Group 1 and Group 2 (based on log rank p-value grouping, Table 4).

Table S3 summarizes hazard ratio for the same drug product KLH/MM for Group S1 relative to Group S2 using Cox PH model. These are computed with RFS as time variable and hospital group (Group S1/ Group S2) as covariate with Group S2 as reference category. For KLH arm the HR value is 1.55, p=0.005 which is statistically significant, implying that patients in hospitals in Group S2 exhibited better response as compared to patients in hospitals in Group S1. For MM arm the corresponding HR value is 0.6, p-value=0.026 which is statistically significant and implying that patients in hospitals in Group S1 exhibited better response as compared to patients in hospitals in Group S2.

Conclusion:

Conclusions provided in the manuscript based on log-rank p-values grouping are also supported by HR grouping analysis. Suggesting grouping methodology has no impact on the final conclusion.
